# Rapid Detection of VREfm Clusters: FTIR Spectroscopy as a Practical Alternative to Whole-Genome Sequencing

**DOI:** 10.1093/ofid/ofaf718

**Published:** 2025-11-24

**Authors:** Jun Hao Wang-Wang, Laia Soler, Elisa Martró, Gemma Clarà, Jessica Hidalgo, Irma Casas, María-José García-Quesada, Montserrat Giménez, Verónica Saludes, Antoni E Bordoy, Pere-Joan Cardona

**Affiliations:** Microbiology Department, Laboratori Clínic Metropolitana Nord, Germans Trias I Pujol University Hospital, Badalona, Spain; Genetics and Microbiology Department, Universitat Autònoma de Barcelona, Bellaterra, Spain; Microbiology Department, Laboratori Clínic Metropolitana Nord, Germans Trias I Pujol University Hospital, Badalona, Spain; Microbiology Department, Laboratori Clínic Metropolitana Nord, Germans Trias I Pujol University Hospital, Badalona, Spain; Genetics and Microbiology Department, Universitat Autònoma de Barcelona, Bellaterra, Spain; Clinical and Experimental Microbiology, Germans Trias i Pujol Research Institute (IGTP), Badalona, Spain; Centro de Investigación Biomédica en Red en Epidemiología y Salud Pública (CIBERESP), Instituto de Salud Carlos III (ISCIII), Madrid, Spain; Microbiology Department, Laboratori Clínic Metropolitana Nord, Germans Trias I Pujol University Hospital, Badalona, Spain; Microbiology Department, Laboratori Clínic Metropolitana Nord, Germans Trias I Pujol University Hospital, Badalona, Spain; Preventive Medicine Department, Germans Trias i Pujol University Hospital, Badalona, Spain; Infection Control Nursing, Infection Control Team, Germans Trias i Pujol University Hospital, Badalona, Spain; NURECARE-IGTP Nursing Research Group, Germans Trias i Pujol Research Institute, Badalona, Spain; Microbiology Department, Laboratori Clínic Metropolitana Nord, Germans Trias I Pujol University Hospital, Badalona, Spain; Centro de Investigación Biomédica en Red en Enfermedades Respiratorias (CIBERES), Instituto de Salud Carlos III (ISCIII), Madrid, Spain; Microbiology Department, Laboratori Clínic Metropolitana Nord, Germans Trias I Pujol University Hospital, Badalona, Spain; Genetics and Microbiology Department, Universitat Autònoma de Barcelona, Bellaterra, Spain; Clinical and Experimental Microbiology, Germans Trias i Pujol Research Institute (IGTP), Badalona, Spain; Centro de Investigación Biomédica en Red en Epidemiología y Salud Pública (CIBERESP), Instituto de Salud Carlos III (ISCIII), Madrid, Spain; Microbiology Department, Laboratori Clínic Metropolitana Nord, Germans Trias I Pujol University Hospital, Badalona, Spain; Clinical and Experimental Microbiology, Germans Trias i Pujol Research Institute (IGTP), Badalona, Spain; Microbiology Department, Laboratori Clínic Metropolitana Nord, Germans Trias I Pujol University Hospital, Badalona, Spain; Genetics and Microbiology Department, Universitat Autònoma de Barcelona, Bellaterra, Spain; Clinical and Experimental Microbiology, Germans Trias i Pujol Research Institute (IGTP), Badalona, Spain; Centro de Investigación Biomédica en Red en Enfermedades Respiratorias (CIBERES), Instituto de Salud Carlos III (ISCIII), Madrid, Spain

**Keywords:** cgSNP, FTIR, outbreak, SKA, vancomycin-resistant enterococcus faecium

## Abstract

Vancomycin-resistant *Enterococcus faecium* (VREfm) has become a significant nosocomial pathogen due to its potential to cause outbreaks. Whole-genome sequencing (WGS) is considered the reference method for determining genomic relatedness among outbreak strains, but its routine use in clinical microbiology laboratories remains challenging. Consequently, faster and simpler typing methods are needed. Fourier transform infrared spectroscopy (FTIR) captures the unique infrared fingerprint of each isolate, enabling the comparison of spectral profiles to infer genomic relatedness. In this study, we evaluated the performance of FTIR for identifying genomic clusters of VREfm in a tertiary hospital, in comparison with three WGS-based methods: core-genome multilocus sequence typing (cgMLST), core-genome single nucleotide polymorphism analysis (cgSNP), and split *k*-mer analysis (SKA). A total of 87 VREfm isolates, collected between April 2020 and October 2023, were typed using both FTIR and WGS. Among these, 56 were associated with three outbreaks in the surgery, nephrology, and oncohematology units, according to conventional epidemiology. Concordance between typing methods was assessed using the Adjusted Rand index (AR) and Adjusted Wallace coefficient (AW). All three WGS-based methods yielded similar clustering results and revealed one monoclonal and two polyclonal outbreaks. Using cgMLST as the reference, an optimal FTIR cutoff range of 0.210–0.227 was determined. FTIR clustering results showed strong concordance with WGS-based methods; however, concordance with SKA was slightly lower. These findings suggest that FTIR provides clustering information comparable to WGS-based methods, providing a rapid and practical alternative to support timely infection control measures during VREfm outbreaks.

Infections caused by vancomycin-resistant *Enterococcus faecium* (VREfm) pose a considerable challenge due to multidrug resistance [1] and their capacity to spread in hospital environments, where they can cause outbreaks [2] and exacerbate the already significant burden of healthcare-associated infections [3]. In 1988, the first vancomycin-resistant *E. faecium* isolates were reported independently in France and the United Kingdom, marking the emergence of this resistance mechanism as a public health concern [4]. In Europe, the proportion of VREfm infections rose from 12.3% (2016) to 19.8% (2023), increasing mortality and costs [5]. The rise in VREfm cases has been particularly noteworthy in Germany [6], where the proportion of VREfm cases reached a peak of 26.3% in 2019 but subsequently declined to 12.7% in 2023 [7]. Conversely, the percentage of VREfm in Spain remained below 3% from 2016 to 2022 [8]. Particularly, in the hospitals of Catalonia, there were no reported cases of VREfm until 2020 when the first cases were detected shortly after the beginning of the COVID-19 pandemic. Currently, the VREfm resistance proportion remains around 3% in Catalonia. At Hospital Germans Trias i Pujol the proportions were 8.5% in 2020, 10.0% in 2021, and 1.0% in 2023.

The 2020–2021 increase in our hospital prompted the evaluation of faster typing tools for outbreak management. DNA-based methods such as pulsed-field gel electrophoresis (PFGE) and whole-genome sequencing (WGS) have been widely used [9, 10], being WGS the current gold standard due to its high discriminatory power [11]. However, routine implementation of WGS remains limited by high turnaround times primarily caused by bioinformatic analysis [12]. A lack of a gold standard WGS analysis approach for VREfm outbreak delimitation also exists. Core genome multilocus sequence typing (cgMLST) has been widely used due to its standardized and stable nomenclature and the availability of commercial software tools [13]. Single nucleotide polymorphism (SNP) distances generated from core genome alignments are also usually used for outbreak delimitation. In this case, some studies have proposed that recombination masking may be inaccurate for highly recombinant species such as *E. faecium,* as it can underestimate the number of SNPs and artificially cluster isolates more closely than they really are [14]. Alternatively, split *k*-mer analysis (SKA) may offer greater discriminatory power than cgMLST for VREfm outbreak delimitation, though current evidence is limited [12, 15]. For these methods, determining the adequate clustering threshold to define close genomic relatedness can also be an important limitation. Therefore, selecting an appropriate WGS-based analysis method for VREfm outbreak analysis requires careful consideration.

Fourier-transform infrared (FTIR) spectroscopy has emerged as a phenotypic alternative with resolution suitable for typing and outbreak delimitation [16]. FTIR quantifies absorption by carbohydrates, lipids, and proteins [17, 18]. Each isolate's infrared fingerprint is then compared with the rest so that relationships between bacterial lineages can be established based on spectrum analysis [19]. Previous work supports FTIR for Gram-positive [2] and Gram-negative pathogens with internal validation [20–23].

The aim of the present study was to evaluate the capacity of IR Biotyper (Bruker GmbH, Leipzig, Germany) to track emergent VREfm genomic clusters at a tertiary hospital in comparison with three different WGS analysis methods.

## MATERIAL AND METHODS

### Study Design and Setting

Retrospective cross-sectional study in a tertiary care hospital serving 200 000 inhabitants and reference for 1 200 000 in Barcelona's Northern Metropolitan Area (Spain). We analyzed 87 VREfm isolates from hospitalized patients (*n* = 83) and four environmental samples from the surgery department, collected April 2020–October 2023. Fifty-six isolates were associated with three epidemiologically defined outbreaks (surgery, nephrology, oncohematology). A nosocomial outbreak was declared by the infection control team upon the detection of ≥2 clinical or ≥3 rectal swab isolates within one month in a single ward. Only isolates from patients who were VREfm negative at the time of admission were considered. Thus, all included isolates were detected after the first 48 hours of hospitalization by routine screening. The date of outbreak closure was set when no VREfm isolates were identified in the same hospital ward for 3 months since the last identification. The remaining isolates were considered unrelated to these outbreaks (Supplementary Table 1).

### Ethics Statement

This study was approved by the Clinical Research Ethics Committee (CEIC) of the University Hospital Germans Trias i Pujol in Barcelona, Spain (PI-24–118).

### Routine Microbiological Diagnostics and Antimicrobial Susceptibility Testing

All isolates were firstly identified with matrix-assisted laser desorption ionization-time of flight mass spectrometry (MALDI-TOF MS, Bruker Daltonik GmbH, Bremen, Germany). The antibiotic susceptibility of vancomycin and teicoplanin was assessed using a gradient test (bioMérieux SA, Marcy-l’Étoile, France), while linezolid susceptibility was evaluated through disk diffusion (Bio-Rad Laboratories Inc., California, United States). For blood cultures, susceptibility to vancomycin, teicoplanin, and linezolid was determined using the VITEK-2 Compact system (bioMérieux SA, Marcy-l’Étoile, France). Interpretation of minimum inhibitory concentration (MIC) values and disk diffusion zone diameters followed the criteria established by the European Committee on Antimicrobial Susceptibility Testing (EUCAST) [24]. All isolates were subsequently preserved in Cryoinstant® Natural storage medium (Scharlab S.L., Barcelona, Spain) at −80°C until further analyses.

### Sample Preparation for FTIR Analysis and Spectrum Analysis

All VREfm isolates were thawed at room temperature and cultured in Columbia Agar + 5% sheep blood (bioMérieux SA, Marcy-l’Étoile, France) for 24 hours at 37°C. Then, bacterial isolates were subcultured in Mueller-Hinton agar medium from a single colony (Becton Dickinson GmbH, Heidelberg, Germany) for another 24 hours at 37°C before testing. Preparation of bacterial suspensions was performed as previously described by Wang-Wang *et al.* [23] Briefly, a loopful of bacterial cells was resuspended in 50 μL of deionized water in a 1.5-mL suspension vial containing metal beads (Bruker GmbH, Leipzig, Germany) and homogenized by vortexing. Subsequently, 50 μL of 70% (v/v) ethanol was added to each vial, followed by a second homogenization step. Then, 15 μL of each bacterial suspension was spotted in quadruplicate onto a 96-spot silicon plate (Bruker GmbH, Leipzig, Germany). For quality control, 12 μL of two infrared test standards (IRTS 1 and IRTS 2, each containing an *Escherichia coli* strain with a defined reference spectrum) were placed in duplicate on the same silicon plate. The plate was then dried at 37°C for 30 minutes prior to insertion into the Biotyper system. For spectrum acquisition, all samples were analyzed in transmission mode using the default analysis settings (wavelength region 1300–800 cm^−1^).

Samples that did not meet the manufacturer's quality criteria were excluded. The acceptance parameters were as follows: absorption between 0.4 and 2, noise (×10^−6^) < 300, signal-to-noise ratio R2 > 200, signal-to-noise ratio R3 > 40, water vapor (×10^−6^) < 300, signal-to-water ratio (R2) > 100, signal-to-water ratio (R3) > 20, and fringes (×10^−6^) < 100. Based on these criteria, 19 samples required reanalysis until at least three valid spectra were obtained for each isolate. Of these, 13 yielded valid spectra, whereas no valid spectra could be recovered for the remaining six samples, which were therefore excluded from the study. An average spectrum for each individual strain was subsequently generated from the qualified spectra using the OPUS 8.2.28 software (Bruker GmbH, Leipzig, Germany).

The resulting average spectra were then used to construct a dendrogram based on hierarchical cluster analysis (HCA) using the Euclidian metric distance and the average linkage method (UPGMA) without dimensionality reduction techniques. The optimal clustering cutoff within a slightly extended range (0.15–0.25) of that recommended by the manufacturer (0.15–0.20) was calculated. Isolates showing an FTIR spectral distance equal to or below this cut-off value were classified within the same FTIR cluster, whereas those exceeding the threshold were considered FTIR singletons.

### Sample Preparation for WGS Analysis and Bioinformatics Analyses

For WGS analysis, all VREfm samples were prepared and sequenced as previously described [23], except for solid sample culture that was performed in Columbia CNA agar (bioMérieux SA, Marcy-l’Étoile, France). The WGS clustering analysis of VREfm isolates was performed based on pairwise genome comparisons using three different genomic approaches: cgMLST, cgSNP, and SKA. Additionally, antimicrobial resistance genes were detected using Abricate v1.0.1 against the NCBI database [25, 26].

Regarding clustering analysis, the observed distributions of pairwise allelic differences and SNPs were used to infer clustering cutoffs. More specifically, for each clustering methodology, the distribution of pairwise distances was modeled applying a Gaussian Mixture Model (GMM) using the Mclust function from the mclust R package (https://www.r-project.org/). The clustering cut-off was then set at the 99th percentile of the fit corresponding to the first component (cgMLST: ≤11 allelic differences; cgSNP: ≤22 SNPs for ST80 and ST80-like isolates, ≤9 SNPs for ST117 isolates; SKA: ≤30 SNPs; Supplementary Fig. 1). For cgMLST analysis, genomic clusters were defined as ≥2 isolates of the same sequence type (ST) and a genetic below the established threshold of allelic differences. cgMLST analysis was performed with Ridom SeqSphere + software 8.5 (Ridom GmbH, Münster, Germany) using SPAdes v3.15.4 for *de novo* assembly, using the typing scheme of 1423 genes published by Been *et al.*, [27] and the parameter “pairwise ignoring missing values”. A quality criterion of ≥97% good cgMLST targets and a coverage of ≥30× was applied. From this analysis, ST, clonal complexes (CC) and complex types (CT) were also obtained. For cgSNP analysis, raw reads were trimmed using Trimmomatic v0.39 (LEADING:3 TRAILING:3 SLIDINGWINDOW:5:25 MINLEN:60) [28]. cgSNP analysis was performed separately for ST80 (and ST80-like) and ST117 isolates to provide a greater degree of pairwise SNP resolution, as recommended [16]. To establish the population structure of the isolates, for each predominant ST, trimmed reads were mapped to a close reference genome when possible, using snippy v4.6.0 (mincov 10, minfrac 0.9); genome alignments without masking for recombination were then used to infer a phylogenetic tree using IQtree v2.2.2.3 [29] with 1000 ultrafast bootstraps and a generalized time-reversible model (GTR). snp-dists was used to calculate pairwise SNP distances. Clusters were defined as ≥2 isolates of the same ST, with a genetic distance below the established SNP threshold according to ST and a monophyletic origin supported by a bootstrap value ≥90%. For SKA analysis, SNPs were defined as “number of split kmers found in both samples where the middle base is an A, C, G, or T but differs between files” [30].

### Concordance Between Clustering Methodologies

Concordance between FTIR and each WGS method was assessed using Adjusted Rand (AR) and Adjusted Wallace (AW) [31–33], using an online tool (www.comparingpartitions.info) with 95% confidence intervals (95% CI). AR indicates the overall agreement of each cluster composition between two techniques. AW measures the directional probability that two isolates detected in a cluster by a reference technique are also detected in the same cluster using an alternative technique.

## RESULTS

### Emergence of VREfm

**Figure 1.**
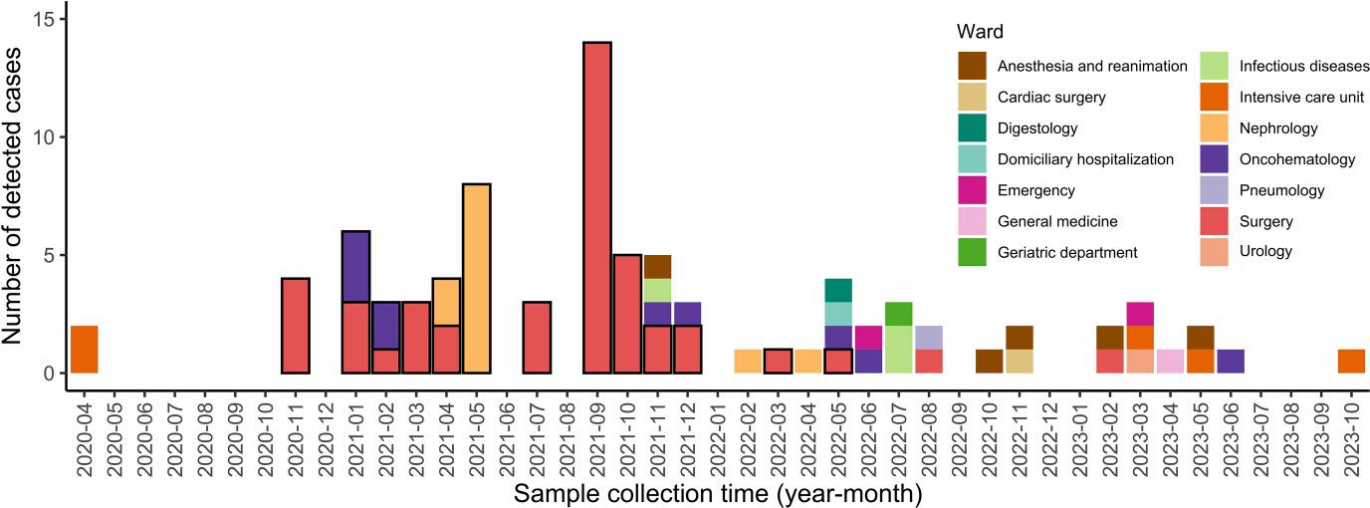
Distribution of VREfm isolates according to their hospital location. Isolates associated with each of the three epidemiological outbreaks (surgery, nephrology and oncohematology wards) are indicated with a black border.

On April 2020, the first VREfm isolates were recovered from rectal swabs of two ICU patients. In November 2020, an outbreak was declared in surgery (three clinical and one surveillance sample). Secondary outbreaks occurred in January and April 2021 in oncohematology and nephrology, respectively. During late 2021, most isolates came from surgery, peaking in September. From February 2022 onward, sporadic cases appeared across wards, mostly not linked to outbreaks (Figure 1, Supplementary Table 1). All isolates showed phenotypic resistance to vancomycin and all but one (isolate 84) susceptibility to linezolid. Regarding teicoplanin, only isolate 87 was sensitive.

### cgMLST and cgSNP Analysis

**Figure 2.**
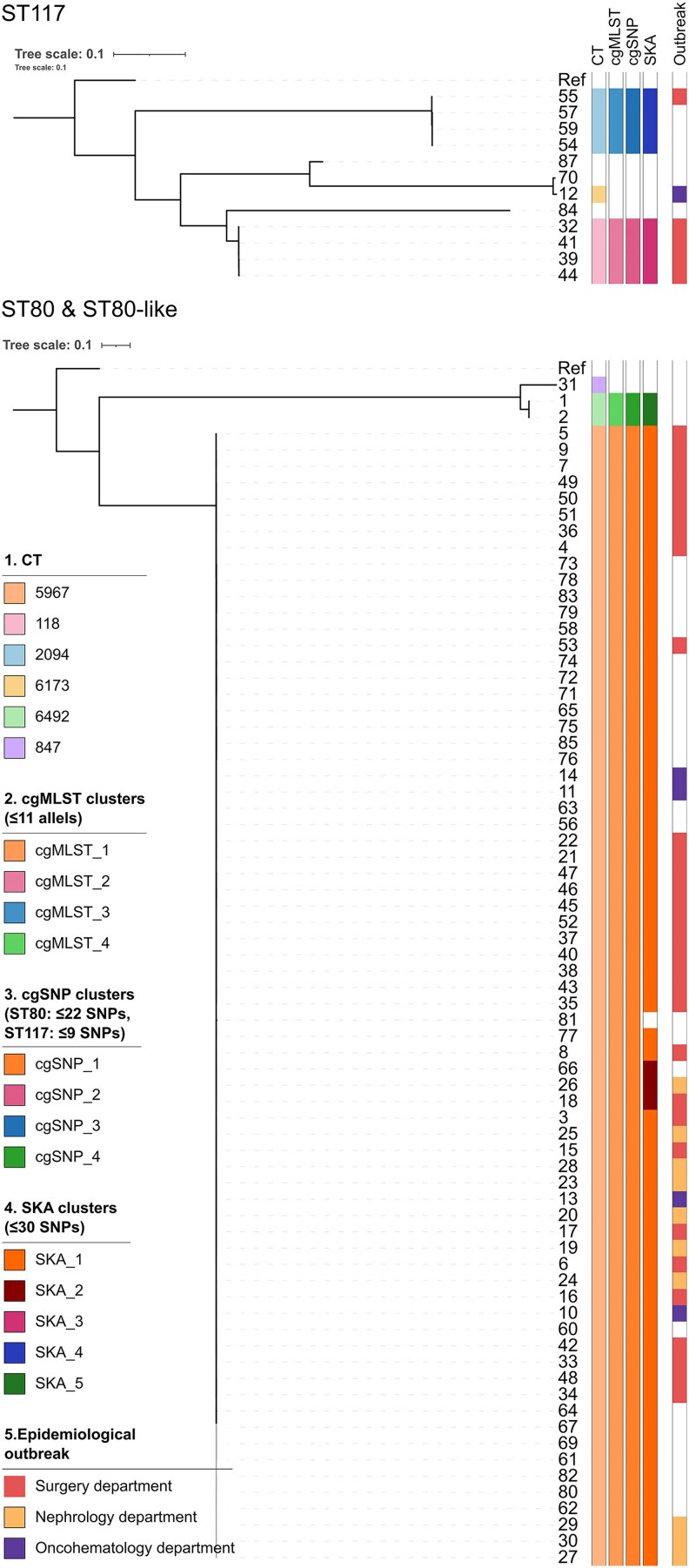
Maximum-likelihood phylogenetic tree (GTR) obtained from cgSNP analysis of VREfm isolates showing clusters identified by cgMLST, cgSNP and SKA approaches as well as epidemiological outbreak. ST80 reference: AUSMDU00004142 (accession no. CP027501.1); ST117 reference: VRE5755 (accession no. DAIGYV000000000.1).

Initial cgMLST analysis using a cutoff of ≤11 allelic differences revealed that most isolates belonged to two differentiated populations: one consisting of ST80 (*n* = 69; of which 66 belonged to CT5967, two to CT6492 and one to CT847) and ST80-like isolates (*n* = 4; three with a novel *gyd* allele and one with a novel *purK* allele; all four belonging to CT5967), and the other comprising isolates of ST117 (*n* = 12). Additionally, one isolate belonged to ST761, and another belonged to novel ST2840 (*atpA* 9, *ddl* 1, *gdh* 1, *purK* 73, *gyd* 12, *pstS* 1, *adk* 1). All typed isolates belonged to clonal complex 17 (CC17). *vanA* was detected in all isolates except 87 (*vanB*); *cfr*(D) was present in isolate 84, matching phenotype. cgMLST grouped all but seven isolates into four clusters (cgMLST_1–cgMLST_4; Figure 2). cgMLST_1 contained 80.5% (70/87), including four environmental isolates, all of which belonged to CT5967. Other clusters had 2–4 isolates each. cgSNP with cutoffs ≤22 (ST80/ST80-like) and ≤9 (ST117) matched cgMLST clustering, identifying four clusters and seven singletons (Figure 2). Of note, the potential effect of not masking recombination sites on the identified clusters was assessed. Masking for recombination consistently resulted in lower pairwise SNP distances than their unmasked counterparts. However, cluster composition after readjusting clustering cut-offs (ie, ≤18 SNPs for ST80 and ST80-like isolates, ≤6 SNPs for ST117 isolates) was identical to that obtained without masking for recombination (Supplementary Figs. 2 and 3). Regarding outbreaks identified by conventional epidemiology, nephrology's outbreak was monoclonal (cgMLST_1), whereas surgery and oncohematology were polyclonal but dominated by cgMLST_1 (Supplementary Table 1).

### SKA Clustering Reveals Little Subdivision Within cgMLST Clusters

SKA identified five clusters (SKA_1–SKA_5) and eight singletons using a cut-off of 30 SNPs. Among the cgMLST-defined clusters, only cgMLST_1 was further subdivided by SKA into two distinct clusters (SKA_1 and SKA_2) and one singleton. SKA_1 was the largest cluster, comprising 75.9% (66/87) of the study isolates, while SKA_2 included the remaining three isolates originally assigned to cgMLST_1 (Figure 2). For all other isolates, identical clustering results between cgMLST and SKA were obtained (AR_cgMLST-SKA_ = 0.850 [0.708–0.998]). No clear epidemiological link existed among SKA_2 isolates; as they were ≥2 months apart and from different wards. Additionally, as the SNP threshold used for SKA clustering considerably differed from those commonly used in the literature (7–12 SNPs) [12–15], a sensitivity analysis was performed on this parameter. Lowering the threshold from 30 to 12 SNPs subdivided cluster SKA_1 into three clusters and one singleton, while not affecting the clustering of the remaining isolates (Supplementary Fig. 4). This resulted in a lower concordance between SKA and cgMLST (AR_cgMLST-SKA12_ = 0.418 [0.260–0.581]). Further decreasing the threshold continued to reduce the concordance between SKA and cgMLST (Supplementary Table 2).

### FTIR Largely Captures WGS Clusters and Reflects Epidemiological Outbreaks

**Figure 3.**
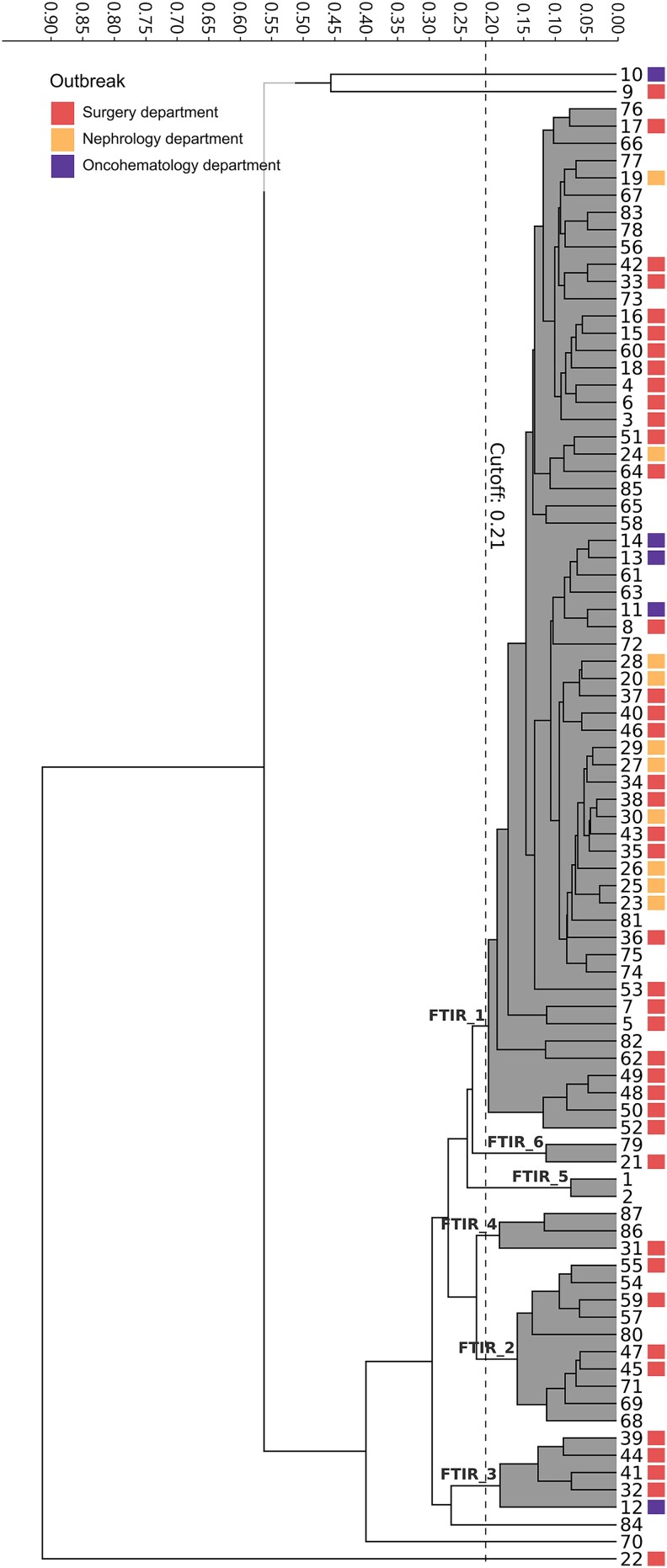
Dendrogram obtained by clustering the FTIR spectra of 87 VREfm. The vertical dashed line indicates the used cutoff value (0.21) for clustering analysis. The resulting FTIR clusters are shadowed in gray. Isolates belonging to epidemiological outbreaks are annotated in color.

**Figure 4.**
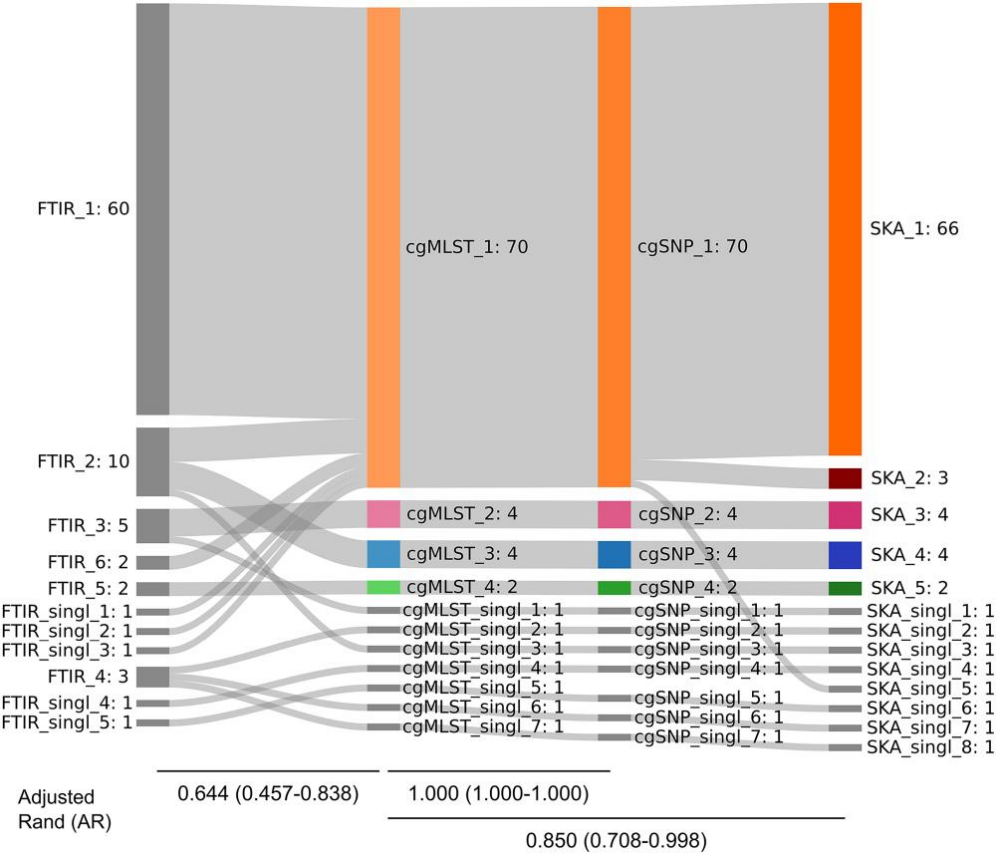
Relationship between Fourier-Transform Infrared spectroscopy (FTIR) and genomic methods (cgMLST, cgSNP, SKA) for genomic clustering of VREfm isolates. Clustering cutoffs (FTIR: ≤0.21, cgMLST: ≤11 allelic differences, cgSNP: ≤22 SNPs for ST80 and ≤9 SNPs for ST117, SKA: ≤30 SNPs). Clusters and singletons detected by each method are indicated with a correlative number along with the number of cases in partition.

Given the similar results obtained across the three WGS-based approaches, and to facilitate interpretation, FTIR clustering performance was first compared with cgMLST as the reference method, and subsequently evaluated against SKA (confusion matrices are provided in Supplementary Tables 3–5). For FTIR clustering, an optimal cutoff range of 0.210 to 0.227 was obtained after maximizing AR (Supplementary Fig. 5) using cgMLST as the reference. Using this cutoff, FTIR yielded six clusters (FTIR_1–FTIR_6) plus five singletons (Figure 3). Cluster cgMLST_1 was largely captured by cluster FTIR_1, which included 60 of the 70 cgMLST_1 isolates (85.7%), including all four environmental isolates. However, seven cgMLST_1 isolates were misclassified by FTIR into clusters FTIR_2 (*n* = 5) and FTIR_6 (*n* = 2), and three were identified as singletons (Figure 4). FTIR_2 also included all four isolates of cgMLST_3 (*n* = 4, ST117) and isolate 68, of ST2840, thus clustering together isolates of different STs. Conversely, full concordance between cluster cgMLST_4 and FTIR_5 (*n* = 2) was observed. Lastly, three out of five cgMLST singletons were grouped together into cluster FTIR_4. Overall, these results yielded a value of 0.644 (95% CI, 0.457–0.838), and 0.944 (95% CI, 0.928–0.960) for AR and AW respectively, when comparing FTIR versus cgMLST (AW_FTIR→cgMLST_) as the reference method. An AW value of 0.489 (95% CI, 0.224–0.754) for cgMLST versus FTIR (AW_cgMLST→FTIR_) as the reference method. The concordance of FTIR with the SKA was slightly lower as SKA further subdivided cluster cgMLST_1 into clusters SKA_1 and SKA_2, and a singleton. Therefore, the comparison of SKA versus FTIR yielded an AR value of 0.543 (95% CI, 0.352–0.745), an AW_FTIR→SKA_ of 0.660 (95% CI, 0.396–0.923) and an AW_SKA→FTIR_ of 0.462 (95% CI, 0.187–0.737). Regarding the three hospital outbreaks, FTIR correctly inferred a true genomic relationship for 33 out of 41 (80.5%) VREfm isolates of the surgery department outbreak: 29 were assigned to cluster FTIR_1 (cgMLST_1) and 4 to cluster FTIR_2 (cgMLST_3); all 10 isolates of the nephrology department outbreak were grouped into FTIR_1 (cgMLST_1); and, for the oncohematology department outbreak, FTIR clustered 3 of the 5 isolates of cluster cgMLST into cluster FTIR_1.

To further investigate the performance of FTIR in different epidemiological scenarios, we divided the cases in two periods: an “outbreak emergence and peak” period from April 2020 to December 2021 (*n* = 60), when most isolates were associated with outbreaks in the surgery, nephrology and oncohematology wards, and a “sporadic cases” period from February 2021 to October 2023 (*n* = 27). The corresponding AR_cgMLST-FTIR_ for the emergence and peak period was 0.686 (95% CI, 0.462–0.919), slightly higher than that of the post-outbreak period, which was 0.562 (95% CI, 0.215–0.938). This suggests that the performance of FTIR might be slightly higher during active outbreak periods.

## DISCUSSION

VREfm is a major cause of a range of infections in humans, including, urinary tract infections, wound infections, bloodstream infections, and endocarditis, and is frequently associated with nosocomial outbreaks. Although our 2020–2021 outbreaks had limited clinical impact, since most cases represented colonization (rectal swabs) rather than infection and no associated fatalities occurred, they demanded substantial infection-control efforts. These included patient isolation, reinforcement of hygiene measures and enhanced environmental disinfection. Four environmental isolates were also recovered, confirming the persistence of VREfm in the hospital environment and supporting targeted cleaning interventions. These measures were guided by typing results, which enabled the rapid identification of transmission clusters and differentiation between unrelated cases, thereby underscoring the importance of rapid and reliable typing methods for outbreak management. Therefore, in the present study, we evaluated FTIR for identifying VREfm genomic clusters. We show that FTIR clusters VREfm isolates in close agreement with multiple WGS approaches, supporting its use as a practical first-line tool for initial screening and outbreak detection to enable faster infection-control responses. In addition to its value for early outbreak detection, FTIR can be particularly useful for ruling out sporadic cases that are not related to previous outbreak strains, thereby avoiding unnecessary investigations and infection control measures. Moreover, identifying sporadic VREfm cases with FTIR can support antimicrobial stewardship programs by indicating elevated antibiotic pressure and facilitating targeted interventions to reduce unnecessary antimicrobial use in those clinical units.

Previous studies have demonstrated the potential of FTIR as a typing tool for Gram-negative bacteria, including *Pseudomonas aeruginosa*, *Klebsiella pneumoniae* and *Acinetobacter baumannii* [20–22]. In line with this, we previously evaluated FTIR for typing *Klebsiella pneumoniae* nosocomial outbreaks, with successful implementation in our hospital setting [23]. More recently, FTIR has also been applied to Gram-positive bacteria, including *Enterococcus faecium*, as demonstrated in recent studies [2, 34, 35]. Here, we provide additional evidence for the expansion of FTIR applicability to outbreak management beyond Gram-negative pathogens.

Most isolates were ST80 and ST117, high-risk clones associated with nosocomial spread [36, 37]. ST80 (83.9% of isolates) predominates in Spain [38], and a recent study in Barcelona reported perfect early-detection concordance between FTIR and genomic methods for an ST80 spread [35]. Although confirmation would be required to determine whether the strains responsible for the outbreaks in our hospital and in the Barcelona study are related, these findings collectively suggest the emergence of ST80 VREfm isolates causing nosocomial outbreaks in this geographic area. Given the rapid dissemination of this ST, continuous surveillance is crucial.

Given the absence of a single gold-standard WGS strategy, we compared cgMLST, cgSNP, and SKA [12, 15]. In the literature, cgMLST analysis has been widely used and is often considered as the standard for outbreak analysis of diverse bacterial species in many settings. cgMLST uses a defined scheme which comprises a large number of conserved genes, which allows for dataset comparison in a standardized manner. Compared with cgMLST, cgSNP analysis offers higher cluster resolution by relying on SNP differences rather than allele distances. However, its performance is highly dependent on the choice of the reference genome. Using a distantly related reference can artificially inflate the number of detected pairwise SNPs, thereby compromising the fine-scale resolution required for accurate outbreak delineation [14]. More recently, SKA has emerged as a robust method for VREfm outbreak investigation, offering higher discriminatory power than cgMLST [12, 15]. Studies by Higgs *et al*. and Maechler *et al.* have shown that SKA more accurately infers patient-to-patient transmission, outperforming cgMLST at commonly used thresholds of ≤25 and ≤20 allelic differences, respectively. At these levels, cgMLST often lacks sufficient resolution to differentiate closely related strains. Despite its strengths, a key challenge in SKA—and in WGS-based approaches in general—is defining appropriate clustering cutoffs. These must be interpreted alongside epidemiological data, which are often complex and context-dependent. In our study, clustering thresholds were selected at the 99th percentile first peak in the global distribution of pairwise allelic or SNP differences and were supported by strong spatiotemporal links among isolates. This approach resulted in a clustering cutoff for cgMLST of ≤11 allelic differences, improving cgMLST resolution compared with commonly used thresholds of ≤25 or ≤20. cgSNP cutoffs differed among STs: ≤22 SNPs for ST80 and ST80-like, and ≤9 SNPs for ST117. Finally, for SKA, a threshold of 30 SNPs was selected—considerably higher than the 7–12 SNP range commonly reported in the literature [12–15]. However, it is important to note that FTIR performance was still acceptable when compared with SKA clustering at a threshold of 12 SNPs. Besides, our tailored approach led to considerable harmonization across the three WGS analyses, in contrast with previous studies where results varied substantially depending on the method and parameters used [12, 15].

In the present study, an AR of 0.644 (95% CI, 0.457–0.838), an AW_FTIR→cgMLST_ of 0.944 (95% CI, 0.928–0.960) and an AW_cgMLST→FTIR_ of 0.489 (95% CI, 0.224–0.754) were obtained. Teng *et al* [16]. suggested that an AW of 0.95 or higher should be met when comparing FTIR versus WGS as the gold standard method. This ensures that FTIR clusters the large majority of isolates clustered by WGS without missing potential transmission events. Additionally, an AW of at least 0.5 should be met when using WGS compared with FTIR as a reference method to avoid detection of falsely clustered isolates, which would trigger unnecessary WGS confirmation. In this work, both AW values were marginally below those proposed by Teng *et al.* A slightly lower concordance was observed between FTIR and SKA clustering (AR 0.543 [95% CI, 0.352–0.745]). This value was similar to the results obtained by Park and Ryoo, who assessed the performance of the FTIR for VREfm clustering in a neonatal intensive care unit outbreak involving four patients. The outbreak was predominantly caused by ST17 and an AR of 0.718 (95% CI, 0.466–0.996) between FTIR and SKA was obtained [39]. In contrast, our study includes three distinct epidemiological outbreaks—one monoclonal and two polyclonal—involving 87 patients, thereby representing a higher level of epidemiological complexity. Thus, our results taken together with those reported by Pitart *et al* [35]. Highlight a potentially broader applicability of FTIR beyond monoclonal outbreak scenarios. It is also important to note that it has been suggested that the performance of FTIR decreases as the dataset size increases [16], which could be partially the cause of the lower performance of FTIR observed in the present study in comparison to the previously published literature. Consequently, further investigation of the performance of FTIR using large datasets of different STs and geographical areas is still needed.

As with WGS, the lack of a standardized clustering cutoff across different laboratories represents a key limitation of FTIR. Before implementing FTIR for early detection of genomic clusters, each laboratory must validate its own clustering cutoff, which may vary based on local epidemiology and the culture media used. The manufacturer recommends a cutoff range of 0.15 to 0.20 for *E. faecium*. Our findings, consistent with previously published data, suggest that the optimal cutoff lies within or near this recommended range [2, 35, 39]. Another limitation of FTIR is its lower discriminatory power relative to WGS-based methods. FTIR provides a global spectral fingerprint that reflects multiple cellular components, including polysaccharides, fatty acids, and proteins. However, the wavelength range applied in our study (1300–800 cm⁻¹) primarily targets the polysaccharide region of the bacterial envelope. Consequently, isolates with identical genomic backgrounds may exhibit differences in their polysaccharide profiles, potentially leading to misclassification [22].

Our study also has several limitations. Firstly, isolates were predominantly ST80 and ST117 and originated from a single hospital. This limited genetic diversity and single-center design restrict the generalizability of our findings, as FTIR performance may differ when applied to other VREfm sequence types, geographic regions, or epidemiological contexts. As FTIR spectral signatures can be influenced by strain-specific phenotypic characteristics, further validation using a more genetically diverse set of isolates is warranted. Expanding FTIR analyses to multicenter collections could not only strengthen methodological validation but also facilitate the early detection of new emerging VREfm lineages, as recently proposed in large-scale FTIR surveillance studies [40]. Secondly, despite the acknowledged inherent differences between genotypic and phenotypic methods, the reasons behind the lack of concordance between FTIR and cgMLST for some isolates were beyond the scope of this study and remain unclear.

Collectively, our results showed that FTIR clustering provides results comparable to those obtained from WGS analyses. While FTIR delivers more limited information, its speed and simplicity make it a viable method for real-time outbreak management. FTIR represents a cost-effective approach (approximately 17€ per sample compared with 70€ for WGS considering only reagents) and does not require highly specialized technical personnel. In terms of turnaround time, WGS results from a positive culture may take up to approximately a week, whereas FTIR can deliver results in about 3 hours.

Based on these findings, we propose a practical workflow for outbreak management. In suspected outbreak situations, FTIR could be used as an initial screening tool to rapidly assess the relatedness of isolates. If FTIR results indicate clonal spread, infection control measures to tackle active transmission should be initiated. However, in the case of epidemiologically linked cases not clustered by FTIR, WGS analysis should be performed to assess genomic relatedness and guide further interventions. Thus, FTIR can serve as a frontline tool for outbreak confirmation in settings where WGS is not immediately accessible, enabling faster and more targeted outbreak responses.

## Supplementary Material

ofaf718_Supplementary_Data

## Supplementary Data

Supplementary materials are available at *Open Forum Infectious Diseases* online. Consisting of data provided by the authors to benefit the reader, the posted materials are not copyedited and are the sole responsibility of the authors, so questions or comments should be addressed to the corresponding author.
