# Supplementary material for: Rapid Detection of VREfm Clusters: FTIR Spectroscopy as a Practical Alternative to Whole-Genome Sequencing
Source: Open Forum Infect Dis. 2025 Nov 24;12(12):ofaf718. doi: 10.1093/ofid/ofaf718 (PMC12687595; doi:10.1093/ofid/ofaf718)
Supplement: ofaf718_Supplementary_Data [file ofaf718_supplementary_data.docx]

**Table S1.** Characteristics of vancomycin-resistant *Enterococcus faecium* isolates and comparison of clustering methodologies.

| **ID** | **Collection date** | **Isolate source** | **Epi. outbreak** | **Hospital location** | **ST** | **CT** | **cgMLST cluster** | **cgSNP** | **SKA cluster** | **FT-IR cluster** |
| --- | --- | --- | --- | --- | --- | --- | --- | --- | --- | --- |
| 1 | 14/04/2020 | RS | No | ICU | 80 | 6492 | cgMLST_4 | cgSNP_4 | SKA_5 | FTIR_5 |
| 2 | 15/04/2020 | RS | No | ICU | 80 | 6492 | cgMLST_4 | cgSNP_4 | SKA_5 | FTIR_5 |
| 3 | 06/11/2020 | BC | SD | SD | 80 | 5967 | cgMLST_1 | cgSNP_1 | SKA_1 | FTIR_1 |
| 4 | 08/11/2020 | WE | SD | SD | 80 | 5967 | cgMLST_1 | cgSNP_1 | SKA_1 | FTIR_1 |
| 5 | 09/11/2020 | WE | SD | SD | 80 | 5967 | cgMLST_1 | cgSNP_1 | SKA_1 | FTIR_1 |
| 6 | 10/11/2020 | RS | SD | SD | 80 | 5967 | cgMLST_1 | cgSNP_1 | SKA_1 | FTIR_1 |
| 7 | 11/01/2021 | RS | SD | SD | 80 | 5967 | cgMLST_1 | cgSNP_1 | SKA_1 | FTIR_1 |
| 8 | 14/01/2021 | RS | SD | SD | 80 | 5967 | cgMLST_1 | cgSNP_1 | SKA_1 | FTIR_1 |
| 9 | 14/01/2021 | RS | SD | SD | 80 | 5967 | cgMLST_1 | cgSNP_1 | SKA_1 | FTIR_singl_1 |
| 10 | 28/01/2021 | RS | OHD | OHD | 80 | 5967 | cgMLST_1 | cgSNP_1 | SKA_1 | FTIR_singl_2 |
| 11 | 28/01/2021 | RS | OHD | OHD | 80 | 5967 | cgMLST_1 | cgSNP_1 | SKA_1 | FTIR_1 |
| 12 | 29/01/2021 | U | OHD | OHD | 117 | 6173 | cgMLST_singl_1 | cgSNP_singl_1 | SKA_singl_1 | FTIR_3 |
| 13 | 11/02/2021 | RS | OHD | OHD | 80 | 5967 | cgMLST_1 | cgSNP_1 | SKA_1 | FTIR_1 |
| 14 | 11/02/2021 | RS | OHD | OHD | 80 | 5967 | cgMLST_1 | cgSNP_1 | SKA_1 | FTIR_1 |
| 15 | 26/02/2021 | U | SD | SD | 80 | 5967 | cgMLST_1 | cgSNP_1 | SKA_1 | FTIR_1 |
| 16 | 08/03/2021 | RS | SD | SD | 80 | 5967 | cgMLST_1 | cgSNP_1 | SKA_1 | FTIR_1 |
| 17 | 09/03/2021 | RS | SD | SD | 80 | 5967 | cgMLST_1 | cgSNP_1 | SKA_1 | FTIR_1 |
| 18 | 16/03/2021 | RS | SD | SD | 80 | 5967 | cgMLST_1 | cgSNP_1 | SKA_2 | FTIR_1 |
| 19 | 06/04/2021 | RS | NPHD | NPHD | 80 | 5967 | cgMLST_1 | cgSNP_1 | SKA_1 | FTIR_1 |
| 20 | 11/04/2021 | RS | NPHD | NPHD | unknown | 5967 | cgMLST_1 | cgSNP_1 | SKA_1 | FTIR_1 |
| 21 | 22/04/2021 | RS | SD | SD | 80 | 5967 | cgMLST_1 | cgSNP_1 | SKA_1 | FTIR_6 |
| 22 | 27/04/2021 | BC | SD | SD | 80 | 5967 | cgMLST_1 | cgSNP_1 | SKA_1 | FTIR_singl_3 |
| 23 | 02/05/2021 | RS | NPHD | NPHD | 80 | 5967 | cgMLST_1 | cgSNP_1 | SKA_1 | FTIR_1 |
| 24 | 13/05/2021 | RS | NPHD | NPHD | 80 | 5967 | cgMLST_1 | cgSNP_1 | SKA_1 | FTIR_1 |
| 25 | 17/05/2021 | RS | NPHD | NPHD | 80 | 5967 | cgMLST_1 | cgSNP_1 | SKA_1 | FTIR_1 |
| 26 | 17/05/2021 | RS | NPHD | NPHD | 80 | 5967 | cgMLST_1 | cgSNP_1 | SKA_2 | FTIR_1 |
| 27 | 17/05/2021 | RS | NPHD | NPHD | 80 | 5967 | cgMLST_1 | cgSNP_1 | SKA_1 | FTIR_1 |
| 28 | 17/05/2021 | RS | NPHD | NPHD | 80 | 5967 | cgMLST_1 | cgSNP_1 | SKA_1 | FTIR_1 |
| 29 | 25/05/2021 | RS | NPHD | NPHD | 80 | 5967 | cgMLST_1 | cgSNP_1 | SKA_1 | FTIR_1 |
| 30 | 25/05/2021 | RS | NPHD | NPHD | 80 | 5967 | cgMLST_1 | cgSNP_1 | SKA_1 | FTIR_1 |
| 31 | 14/07/2021 | RS | SD | SD | 80 | 847 | cgMLST_singl_2 | cgSNP_singl_2 | SKA_singl_2 | FTIR_4 |
| 32 | 18/07/2021 | BC | SD | SD | 117 | 118 | cgMLST_2 | cgSNP_2 | SKA_3 | FTIR_3 |
| 33 | 27/07/2021 | RS | SD | SD | 80 | 5967 | cgMLST_1 | cgSNP_1 | SKA_1 | FTIR_1 |
| 34 | 03/09/2021 | BC | SD | SD | 80 | 5967 | cgMLST_1 | cgSNP_1 | SKA_1 | FTIR_1 |
| 35 | 05/09/2021 | RS | SD | SD | 80 | 5967 | cgMLST_1 | cgSNP_1 | SKA_1 | FTIR_1 |
| 36 | 07/09/2021 | RS | SD | SD | 80 | 5967 | cgMLST_1 | cgSNP_1 | SKA_1 | FTIR_1 |
| 37 | 07/09/2021 | RS | SD | SD | 80 | 5967 | cgMLST_1 | cgSNP_1 | SKA_1 | FTIR_1 |
| 38 | 07/09/2021 | RS | SD | SD | 80 | 5967 | cgMLST_1 | cgSNP_1 | SKA_1 | FTIR_1 |
| 39 | 07/09/2021 | RS | SD | SD | 117 | 118 | cgMLST_2 | cgSNP_2 | SKA_3 | FTIR_3 |
| 40 | 13/09/2021 | RS | SD | SD | 80 | 5967 | cgMLST_1 | cgSNP_1 | SKA_1 | FTIR_1 |
| 41 | 13/09/2021 | RS | SD | SD | 117 | 118 | cgMLST_2 | cgSNP_2 | SKA_3 | FTIR_3 |
| 42 | 13/09/2021 | RS | SD | SD | 80 | 5967 | cgMLST_1 | cgSNP_1 | SKA_1 | FTIR_1 |
| 43 | 13/09/2021 | RS | SD | SD | 80 | 5967 | cgMLST_1 | cgSNP_1 | SKA_1 | FTIR_1 |
| 44 | 14/09/2021 | RS | SD | SD | 117 | 118 | cgMLST_2 | cgSNP_2 | SKA_3 | FTIR_3 |
| 45 | 29/09/2021 | RS | SD | SD | 80 | 5967 | cgMLST_1 | cgSNP_1 | SKA_1 | FTIR_2 |
| 46 | 30/09/2021 | RS | SD | SD | 80 | 5967 | cgMLST_1 | cgSNP_1 | SKA_1 | FTIR_1 |
| 47 | 30/09/2021 | RS | SD | SD | 80 | 5967 | cgMLST_1 | cgSNP_1 | SKA_1 | FTIR_2 |
| 48 | 05/10/2021 | EI | SD | SD | 80 | 5967 | cgMLST_1 | cgSNP_1 | SKA_1 | FTIR_1 |
| 49 | 05/10/2021 | EI | SD | SD | 80 | 5967 | cgMLST_1 | cgSNP_1 | SKA_1 | FTIR_1 |
| 50 | 05/10/2021 | EI | SD | SD | 80 | 5967 | cgMLST_1 | cgSNP_1 | SKA_1 | FTIR_1 |
| 51 | 05/10/2021 | EI | SD | SD | 80 | 5967 | cgMLST_1 | cgSNP_1 | SKA_1 | FTIR_1 |
| 52 | 25/10/2021 | RS | SD | SD | 80 | 5967 | cgMLST_1 | cgSNP_1 | SKA_1 | FTIR_1 |
| 53 | 02/11/2021 | RS | SD | SD | 80 | 5967 | cgMLST_1 | cgSNP_1 | SKA_1 | FTIR_1 |
| 54 | 08/11/2021 | RS | No | ARD | 117 | 2094 | cgMLST_3 | cgSNP_3 | SKA_4 | FTIR_2 |
| 55 | 15/11/2021 | RS | SD | SD | 117 | 2094 | cgMLST_3 | cgSNP_3 | SKA_4 | FTIR_2 |
| 56 | 16/11/2021 | RS | No | IDD | 80 | 5967 | cgMLST_1 | cgSNP_1 | SKA_1 | FTIR_1 |
| 57 | 22/11/2021 | RS | No | OHD | 117 | 2094 | cgMLST_3 | cgSNP_3 | SKA_4 | FTIR_2 |
| 58 | 07/12/2021 | RS | No | OHD | 80 | 5967 | cgMLST_1 | cgSNP_1 | SKA_1 | FTIR_1 |
| 59 | 15/12/2021 | RS | SD | SD | 117 | 2094 | cgMLST_3 | cgSNP_3 | SKA_4 | FTIR_2 |
| 60 | 16/12/2021 | RS | SD | SD | 80 | 5967 | cgMLST_1 | cgSNP_1 | SKA_1 | FTIR_1 |
| 61 | 03/02/2022 | RS | No | NPHD | 80 | 5967 | cgMLST_1 | cgSNP_1 | SKA_1 | FTIR_1 |
| 62 | 09/03/2022 | RS | SD | SD | 80 | 5967 | cgMLST_1 | cgSNP_1 | SKA_1 | FTIR_1 |
| 63 | 20/04/2022 | RS | No | NPHD | 80 | 5967 | cgMLST_1 | cgSNP_1 | SKA_1 | FTIR_1 |
| 64 | 02/05/2022 | RS | SD | SD | 80 | 5967 | cgMLST_1 | cgSNP_1 | SKA_1 | FTIR_1 |
| 65 | 04/05/2022 | RS | No | OHD | 80 | 5967 | cgMLST_1 | cgSNP_1 | SKA_1 | FTIR_1 |
| 66 | 26/05/2022 | RS | No | DCD | 80 | 5967 | cgMLST_1 | cgSNP_1 | SKA_2 | FTIR_1 |
| 67 | 27/05/2022 | WE | No | DD | 80 | 5967 | cgMLST_1 | cgSNP_1 | SKA_1 | FTIR_1 |
| 68 | 13/06/2022 | RS | No | OHD | 2840 | unknown | cgMLST_singl_3 | cgSNP_singl_3 | SKA_singl_3 | FTIR_2 |
| 69 | 20/06/2022 | RS | No | ED | 80 | 5967 | cgMLST_1 | cgSNP_1 | SKA_1 | FTIR_2 |
| 70 | 01/07/2022 | RS | No | IDD | 117 | unknown | cgMLST_singl_4 | cgSNP_singl_4 | SKA_singl_4 | FTIR_singl_4 |
| 71 | 08/07/2022 | RS | No | IDD | 80 | 5967 | cgMLST_1 | cgSNP_1 | SKA_1 | FTIR_2 |
| 72 | 12/07/2022 | RS | No | GMD | 80 | 5967 | cgMLST_1 | cgSNP_1 | SKA_1 | FTIR_1 |
| 73 | 23/08/2022 | RS | No | SD | 80 | 5967 | cgMLST_1 | cgSNP_1 | SKA_1 | FTIR_1 |
| 74 | 23/08/2022 | RS | No | PD | 80 | 5967 | cgMLST_1 | cgSNP_1 | SKA_1 | FTIR_1 |
| 75 | 10/10/2022 | RS | No | ARD | unknown | 5967 | cgMLST_1 | cgSNP_1 | SKA_1 | FTIR_1 |
| 76 | 15/11/2022 | RS | No | CCU | unknown | 5967 | cgMLST_1 | cgSNP_1 | SKA_1 | FTIR_1 |
| 77 | 24/11/2022 | RS | No | ARD | 80 | 5967 | cgMLST_1 | cgSNP_1 | SKA_1 | FTIR_1 |
| 78 | 03/02/2023 | WE | No | SD | 80 | 5967 | cgMLST_1 | cgSNP_1 | SKA_1 | FTIR_1 |
| 79 | 21/02/2023 | RS | No | ARD | 80 | 5967 | cgMLST_1 | cgSNP_1 | SKA_1 | FTIR_6 |
| 80 | 20/03/2023 | RS | No | UD | 80 | 5967 | cgMLST_1 | cgSNP_1 | SKA_1 | FTIR_2 |
| 81 | 20/03/2023 | RS | No | ED | 80 | 5967 | cgMLST_1 | cgSNP_1 | SKA_singl_5 | FTIR_1 |
| 82 | 23/03/2023 | RS | No | ICU | 80 | 5967 | cgMLST_1 | cgSNP_1 | SKA_1 | FTIR_1 |
| 83 | 04/04/2023 | RS | No | GMD | 80 | 5967 | cgMLST_1 | cgSNP_1 | SKA_1 | FTIR_1 |
| 84 | 12/05/2023 | RS | No | ICU | 117 | unknown | cgMLST_singl_5 | cgSNP_singl_5 | SKA_singl_6 | FTIR_singl_5 |
| 85 | 19/05/2023 | RS | No | ARD | unknown | 5967 | cgMLST_1 | cgSNP_1 | SKA_1 | FTIR_1 |
| 86 | 16/06/2023 | RS | No | OHD | 761 | unknown | cgMLST_singl_6 | cgSNP_singl_6 | SKA_singl_7 | FTIR_4 |
| 87 | 03/10/2023 | RS | No | ICU | 117 | unknown | cgMLST_singl_7 | cgSNP_singl_7 | SKA_singl_8 | FTIR_4 |

ARD: Anesthesia and reanimation department, BC: Blood culture, CCU: Cardiac care unit, DCD: Domiciliary care department, DD: Digestology department, ED: Emergency department, EI: Environmental isolate, GMD: General medicine department, ICU: Intensive care unit, IDD: Infectious disease department, NPHD: Nephrology department, OHD: Oncohematology department, PD: Pneumology department, RS: Rectal swab, SD: Surgery department, ST: Sequence type, U: Urine, UD: Urology department, WE: Wound exudate.

**Table S2.** Adjusted Rand and jackknife pseudo-values 95% CI for all clustering methods used. Clustering cut-off are indicated in parenthesis.

|  | **cgMLST cluster (≤11 allelic diff.)** | **cgSNP (≤22/9 SNPs)** | **SKA cluster (≤30 SNPs)** | **SKA cluster (≤12 SNPs)** | **SKA cluster (≤10 SNPs)** | **SKA cluster (≤7 SNPs)** |
| --- | --- | --- | --- | --- | --- | --- |
| **cgSNP (≤22/9 SNPs)*** | 1.000 (1.000-1.000) |  |  |  |  |  |
| **SKA cluster (≤30 SNPs)** | 0.850 (0.708-0.998) | 0.850 (0.708-0.998) |  |  |  |  |
| **SKA cluster (≤12 SNPs)** | 0.418 (0.260-0.581) | 0.418 (0.260-0.581) | 0.526 (0.371-0.687) |  |  |  |
| **SKA cluster (≤10 SNPs)** | 0.347 (0.202-0.496) | 0.347 (0.202-0.496) | 0.443 (0.293-0.596) | 0.886 (0.783-0.992) |  |  |
| **SKA cluster (≤7 SNPs)** | 0.244 (0.125-0.365) | 0.244 (0.125-0.365) | 0.316 (0.185-0.450) | 0.689 (0.527-0.856) | 0.796 (0.655-0.941) |  |
| **FT-IR cluster**  **(0.210–0.227)** | 0.644 (0.457-0.838) | 0.644 (0.457-0.838) | 0.543 (0.352-0.745) | 0.283 (0.125-0.447) | 0.218 (0.074-0.367) | 0.180 (0.054-0.309) |
| * ≤22 SNPs for ST80 and ST80-like isolates, ≤9 SNPs for ST117 isolates | | | | | | |

**Table S3.** Confusion matrix comparing FTIR vs. cgMLST clusters.

|  | **FTIR_1** | **FTIR_2** | **FTIR_3** | **FTIR_4** | **FTIR_5** | **FTIR_6** | **FTIR_singl_1** | **FTIR_singl_2** | **FTIR_singl_3** | **FTIR_singl_4** | **FTIR_singl_5** | **Total** |
| --- | --- | --- | --- | --- | --- | --- | --- | --- | --- | --- | --- | --- |
| **cgMLST_1** | 60 | 5 |  |  |  | 2 | 1 | 1 | 1 |  |  | 70 |
| **cgMLST_2** |  |  | 4 |  |  |  |  |  |  |  |  | 4 |
| **cgMLST_3** |  | 4 |  |  |  |  |  |  |  |  |  | 4 |
| **cgMLST_4** |  |  |  |  | 2 |  |  |  |  |  |  | 2 |
| **cgMLST_singl_1** |  |  | 1 |  |  |  |  |  |  |  |  | 1 |
| **cgMLST_singl_2** |  |  |  | 1 |  |  |  |  |  |  |  | 1 |
| **cgMLST_singl_3** |  | 1 |  |  |  |  |  |  |  |  |  | 1 |
| **cgMLST_singl_4** |  |  |  |  |  |  |  |  |  | 1 |  | 1 |
| **cgMLST_singl_5** |  |  |  |  |  |  |  |  |  |  | 1 | 1 |
| **cgMLST_singl_6** |  |  |  | 1 |  |  |  |  |  |  |  | 1 |
| **cgMLST_singl_7** |  |  |  | 1 |  |  |  |  |  |  |  | 1 |
| **Total** | 60 | 10 | 5 | 3 | 2 | 2 | 1 | 1 | 1 | 1 | 1 | 87 |

**Table S4.** Confusion matrix comparing FTIR vs. cgSNP clusters.

|  | **FTIR_1** | **FTIR_2** | **FTIR_3** | **FTIR_4** | **FTIR_5** | **FTIR_6** | **FTIR_singl_1** | **FTIR_singl_2** | **FTIR_singl_3** | **FTIR_singl_4** | **FTIR_singl_5** | **Total** |
| --- | --- | --- | --- | --- | --- | --- | --- | --- | --- | --- | --- | --- |
| **cgSNP_1** | 60 | 5 |  |  |  | 2 | 1 | 1 | 1 |  |  | 70 |
| **cgSNP_2** |  |  | 4 |  |  |  |  |  |  |  |  | 4 |
| **cgSNP_3** |  | 4 |  |  |  |  |  |  |  |  |  | 4 |
| **cgSNP_4** |  |  |  |  | 2 |  |  |  |  |  |  | 2 |
| **cgSNP_singl_1** |  |  | 1 |  |  |  |  |  |  |  |  | 1 |
| **cgSNP_singl_2** |  |  |  | 1 |  |  |  |  |  |  |  | 1 |
| **cgSNP_singl_3** |  | 1 |  |  |  |  |  |  |  |  |  | 1 |
| **cgSNP_singl_4** |  |  |  |  |  |  |  |  |  | 1 |  | 1 |
| **cgSNP_singl_5** |  |  |  |  |  |  |  |  |  |  | 1 | 1 |
| **cgSNP_singl_6** |  |  |  | 1 |  |  |  |  |  |  |  | 1 |
| **cgSNP_singl_7** |  |  |  | 1 |  |  |  |  |  |  |  | 1 |
| **Total** | 60 | 10 | 5 | 3 | 2 | 2 | 1 | 1 | 1 | 1 | 1 | 87 |

**Table S5.** Confusion matrix comparing FTIR vs. SKA clusters.

|  | **FTIR_1** | **FTIR_2** | **FTIR_3** | **FTIR_4** | **FTIR_5** | **FTIR_6** | **FTIR_singl_1** | **FTIR_singl_2** | **FTIR_singl_3** | **FTIR_singl_4** | **FTIR_singl_5** | **Total** |
| --- | --- | --- | --- | --- | --- | --- | --- | --- | --- | --- | --- | --- |
| **SKA_1** | 56 | 5 |  |  |  | 2 | 1 | 1 | 1 |  |  | 66 |
| **SKA_2** | 3 |  |  |  |  |  |  |  |  |  |  | 3 |
| **SKA_3** |  |  | 4 |  |  |  |  |  |  |  |  | 4 |
| **SKA_4** |  | 4 |  |  |  |  |  |  |  |  |  | 4 |
| **SKA_5** |  |  |  |  | 2 |  |  |  |  |  |  | 2 |
| **SKA_singl_1** |  |  | 1 |  |  |  |  |  |  |  |  | 1 |
| **SKA_singl_2** |  |  |  | 1 |  |  |  |  |  |  |  | 1 |
| **SKA_singl_3** |  | 1 |  |  |  |  |  |  |  |  |  | 1 |
| **SKA_singl_4** |  |  |  |  |  |  |  |  |  | 1 |  | 1 |
| **SKA_singl_5** | 1 |  |  |  |  |  |  |  |  |  |  | 1 |
| **SKA_singl_6** |  |  |  |  |  |  |  |  |  |  | 1 | 1 |
| **SKA_singl_7** |  |  |  | 1 |  |  |  |  |  |  |  | 1 |
| **SKA_singl_8** |  |  |  | 1 |  |  |  |  |  |  |  | 1 |
| **Total** | 60 | 10 | 5 | 3 | 2 | 2 | 1 | 1 | 1 | 1 | 1 | 87 |


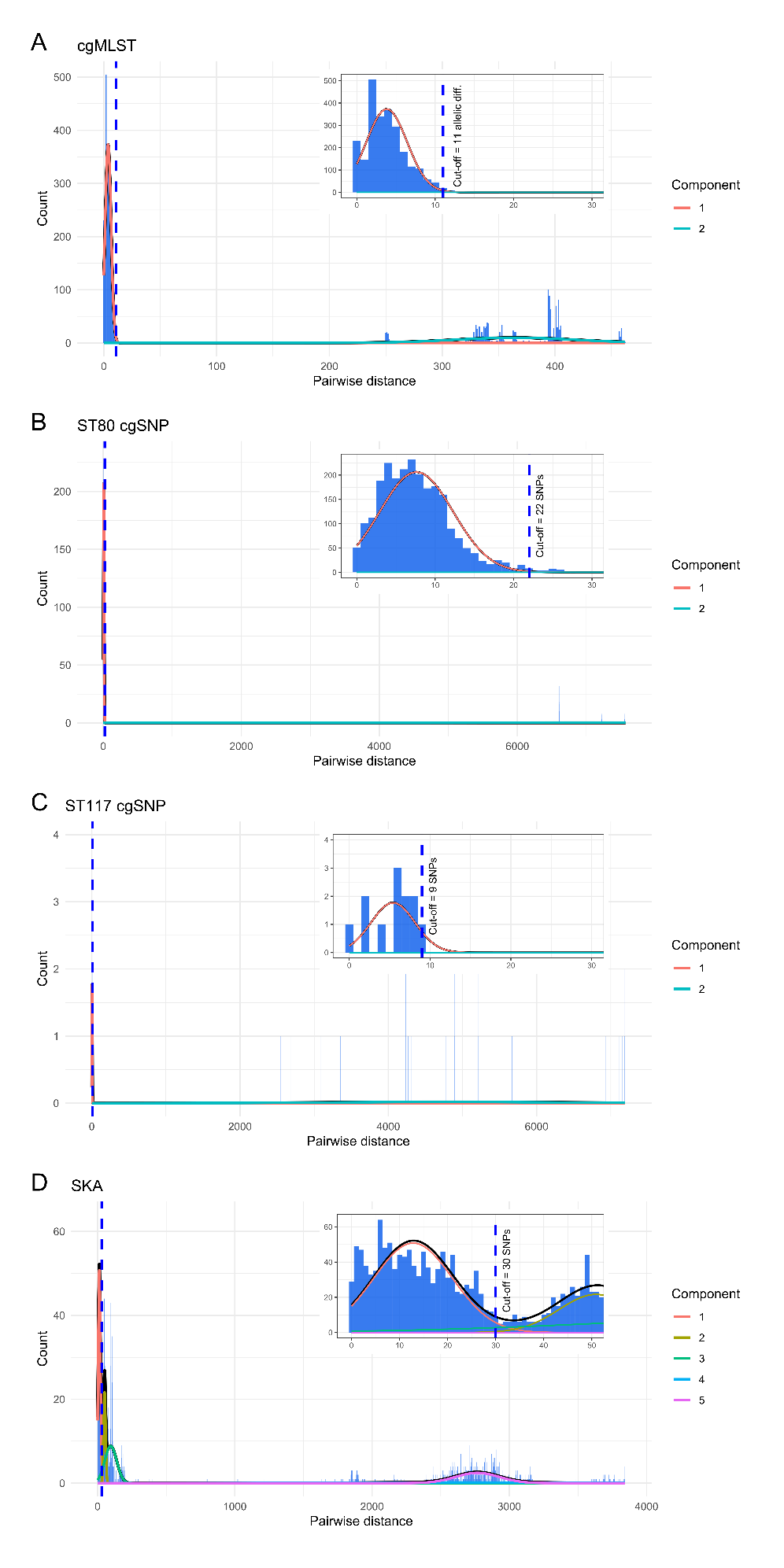


**Figure S1.** Detail of the global distribution of pairwise distances for each WGS analysis used. Dashed blue lines indicate the thresholds applied, which corresponded to the 99^th^ percentile of the first mode in the distribution.


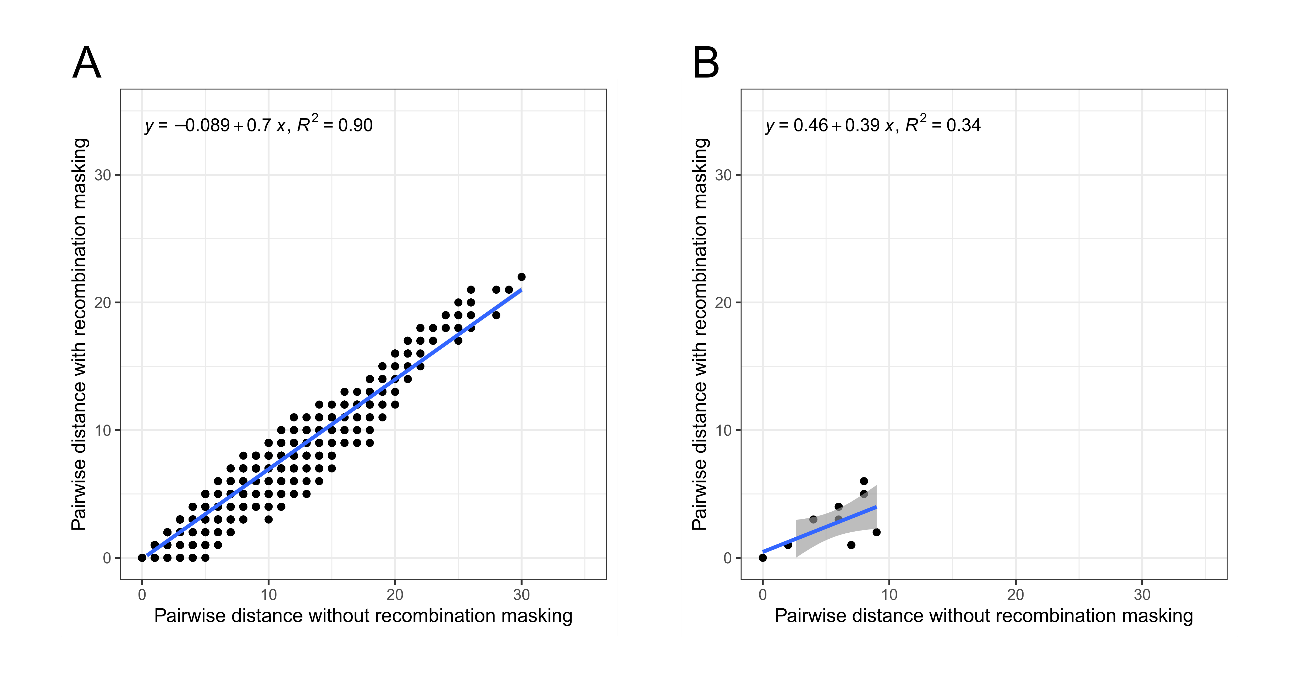


**Figure S2.** Relationship between pairwise SNP distances before and after masking for recombination. The blue line represents the line of best fit using a linear regression model (y ~ x) with the 95% confidence interval around the smoothing function shown in grey. (A) ST80 isolates, (B) ST117 isolates. In both panels x and y axis limits have been restricted to a maximum pairwise distance of ≤35 SNPs.


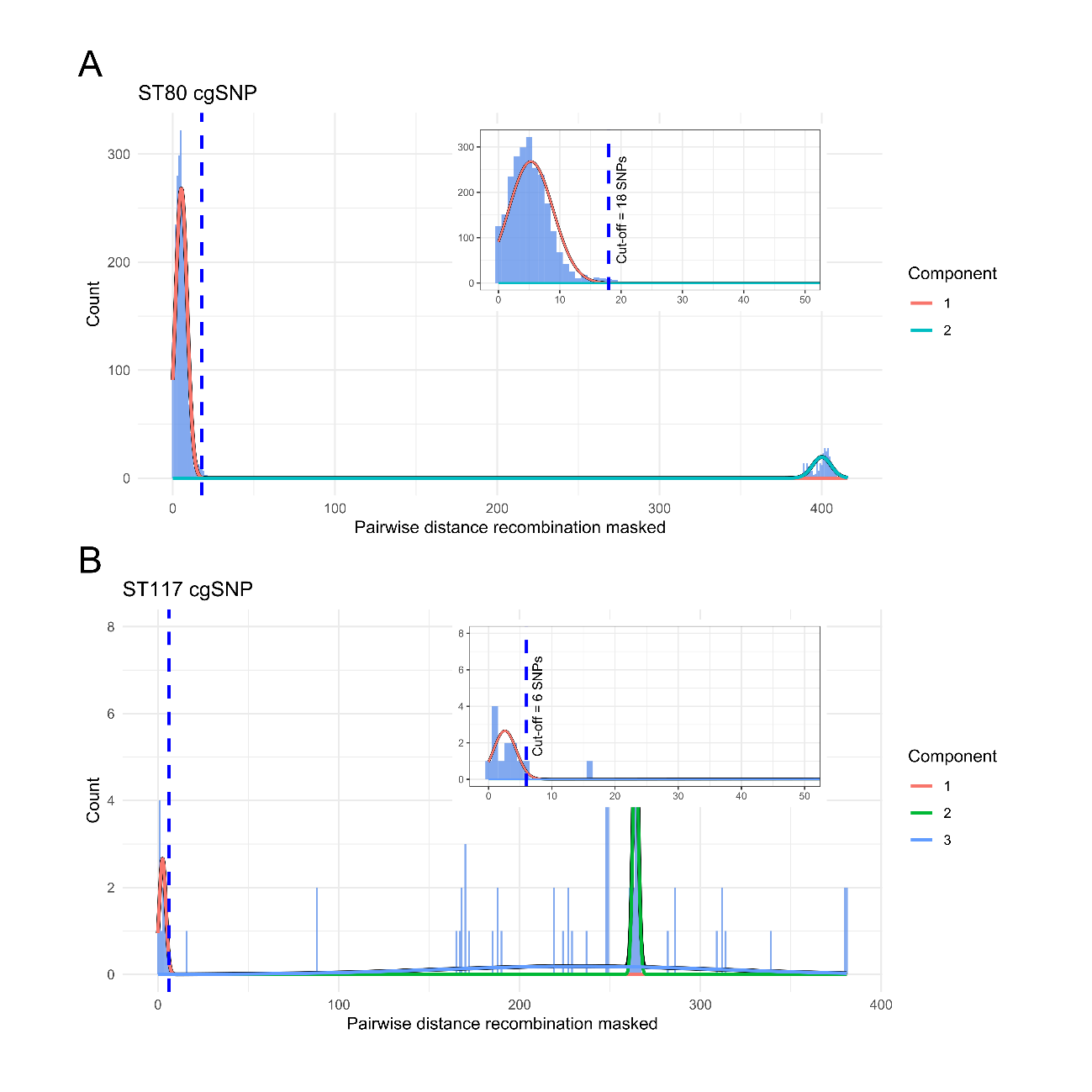


**Figure S3.** Detail of the global distribution of pairwise distances for cgSNP analysis masking for recombination. Dashed blue lines indicate the thresholds applied, which corresponded to the 99^th^ percentile of the first mode in the distribution.


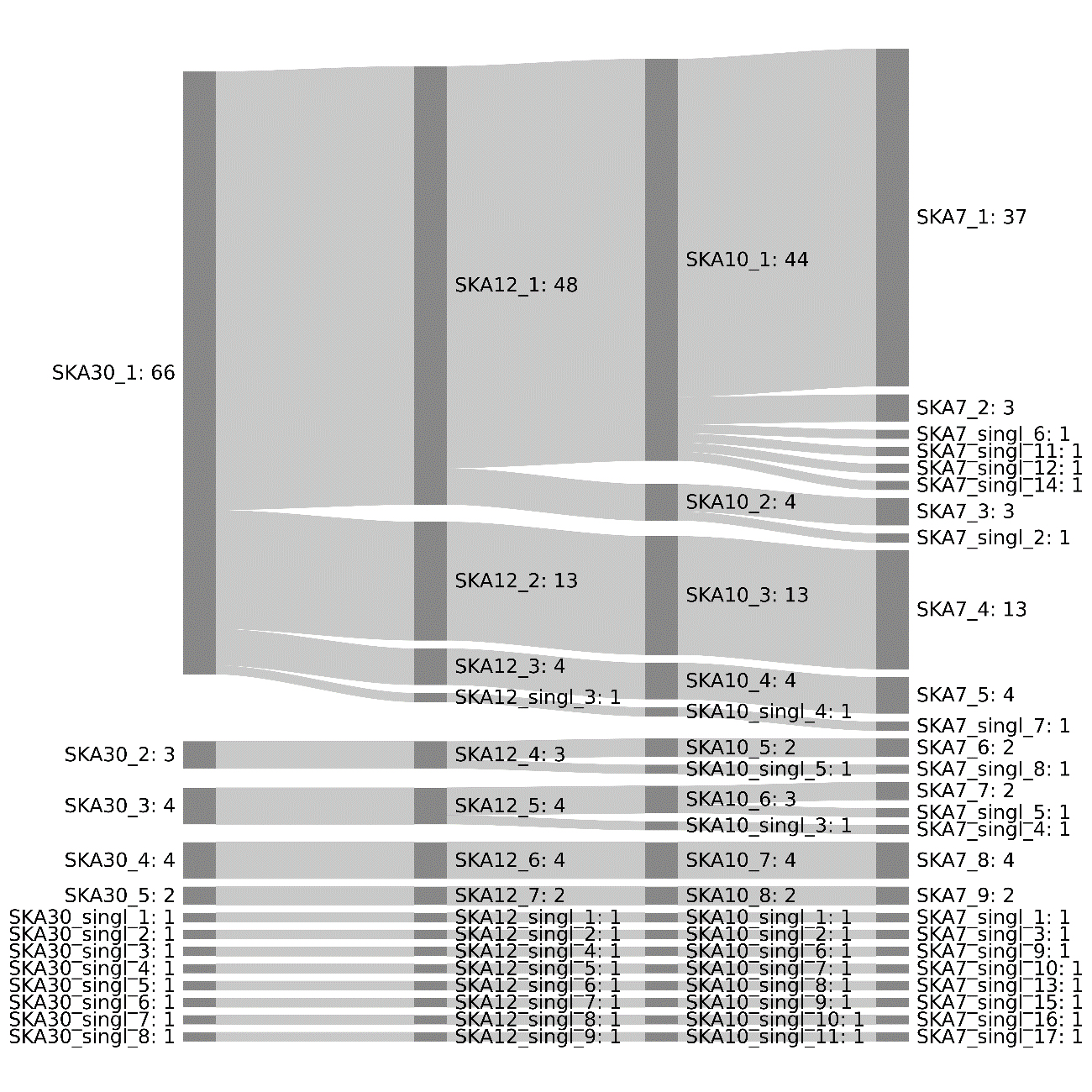


**Figure S4.** Clustering at different SKA thresholds (30, 12, 10, and 7 SNPs). Clusters and singletons detected by at each threshold are indicated with a correlative number along with the number of cases in partition.


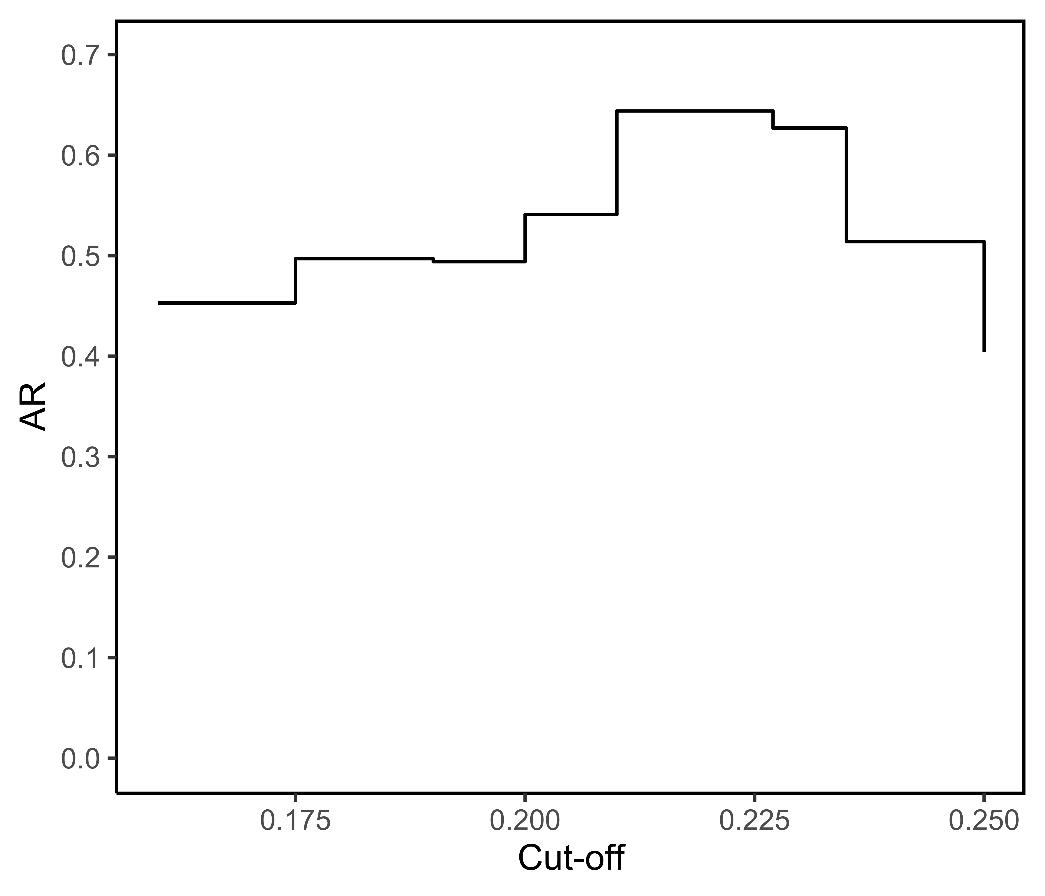


**Figure S5.** FTIR clustering cut-off value. The 0.210 to 0.227 cut-off range maximized the Adjusted Rand index (AR) for the clustering of the study dataset (N=87) considering cgMLST as the reference method. A slightly extended range (0.15-0.25) of that recommended by the manufacturer (0.15-0.20) was evaluated.
